# The modifying effect of dietary index for gut microbiota on the association between urinary arsenic exposure and bladder cancer risk: a nationwide cohort study

**DOI:** 10.3389/fnut.2025.1723496

**Published:** 2025-12-03

**Authors:** Lingfeng Sun, Chengyi Liu

**Affiliations:** Department of Urology, LU’AN Hospital of Anhui Medical University, Lu’an, Anhui, China

**Keywords:** arsenic, bladder cancer, DIGM, dietary index, effect modification, NHANES

## Abstract

**Objective:**

Arsenic exposure is a well-established risk factor for bladder cancer, but substantial individual variability in susceptibility suggests the potential role of effect modifiers. The gut microbiome, which is influenced by diet, may regulate arsenic metabolism and toxicity. The Dietary Index for Gut Microbiota (DIGM) quantifies the potential of diet to foster a beneficial gut ecosystem, yet its role in modifying arsenic-related carcinogenesis remains unclear. This study aimed to investigate the potential interaction between urinary arsenic levels and DIGM on bladder cancer risk.

**Methods:**

We conducted a cross-sectional analysis using data from the National Health and Nutrition Examination Survey (NHANES) 2007–2018. Bladder cancer status was obtained through self-reported medical questionnaires. Total urinary arsenic was measured using ICP-DRC-MS, and arsenobetaine was subtracted to estimate toxic inorganic arsenic exposure. DIGM was constructed based on 24-h dietary recall data. Weighted multivariable logistic regression models were used to evaluate associations, and interaction was tested by including a product term (urinary arsenic*DIGM) and assessed on both additive and multiplicative scales.

**Results:**

Among 4,889 participants (mean age 47.47 years; 50.34% male), 585 had bladder cancer. After adjusting for covariates, elevated urinary arsenic was associated with increased bladder cancer risk (OR: 2.22, 95% CI: 1.53–3.22). Interaction analysis revealed a significant multiplicative interaction between urinary arsenic and DIGM (P-interaction = 0.022). Stratified analysis showed a strong positive association between urinary arsenic and bladder cancer in participants in the lowest DIGM tertile (OR: 1.95, 95% CI: 1.46–2.61). This association was significantly attenuated and became non-significant in those in the highest DIGM tertile (OR: 1.13, 95% CI: 0.89–1.44). Measures of additive interaction (RERI = 0.47, AP = 0.31) further supported a synergistic effect.

**Conclusion:**

Our findings suggest that a diet promoting a healthier gut microbiota, as indicated by a higher DIGM score, may attenuate the association between urinary arsenic exposure and bladder cancer risk. These results indicate that dietary intervention could be a strategic approach to mitigate urinary arsenic-related cancer risk, highlighting the importance of diet–gut–microbiome interactions in environmental carcinogenesis.

## 1 Introduction

Bladder cancer remains a significant global public health burden, ranking as the tenth most common cancer with approximately 573,000 new cases and 213,000 deaths annually (1). Established risk factors include tobacco smoking, occupational exposure to aromatic amines, and significant exposure to environmental carcinogens such as arsenic (2). The carcinogenicity of inorganic arsenic, particularly its association with urothelial carcinoma, has been well-demonstrated in epidemiological studies of highly exposed populations in Bangladesh, Chile, and Taiwan (3, 4). Based on sufficient evidence from human studies, the International Agency for Research on Cancer has classified arsenic and arsenic compounds as Group 1 carcinogens (5).

The primary route of human exposure to inorganic arsenic is through contaminated drinking water, which affects millions of people worldwide (6). However, dietary sources, particularly rice and other grains, also contribute significantly to arsenic exposure in many populations (7). After absorption, inorganic arsenic undergoes complex biotransformation processes, including reductive and oxidative methylation, producing monomethylated and dimethylated metabolites that are primarily excreted in urine (8). The efficiency of this methylation process varies considerably among individuals and has been identified as a determinant of susceptibility to arsenic-related health effects, including cancer (9). Although the association between arsenic exposure and bladder cancer risk is well-established, significant interindividual variability in susceptibility cannot be fully explained by differences in exposure levels or methylation capacity (10). This variability suggests the involvement of other modifying factors, including nutritional status and dietary patterns (11).

Emerging evidence indicates that the gut microbiome plays a crucial role in the metabolism of xenobiotics, including the biotransformation of environmental toxicants such as arsenic (12). The composition and functional capacity of the gut microbiota can influence the metabolic fate of arsenic, potentially altering its toxicity and excretion patterns (13).

Diet is the most important modifiable factor shaping the gut microbial ecosystem (14). Specific dietary components, including dietary fiber, polyphenols, and fermented foods, can promote the abundance of beneficial bacterial taxa and enhance microbial diversity (15). Such dietary patterns have been associated with improved metabolic health and reduced inflammation, which may mitigate carcinogenic processes (16). Conversely, Western dietary patterns characterized by high consumption of processed meats, saturated fats, and refined carbohydrates may promote dysbiosis and increase susceptibility to environmental carcinogens (17). The DIGM is a novel scoring system designed to quantify the potential of an individual’s diet to promote a beneficial gut microbial profile (18). This index integrates current scientific understanding of dietary components that influence microbial composition and function, providing a comprehensive measure of die-gut microbiome interactions (19). However, whether DIGM can modify the relationship between arsenic exposure and bladder cancer risk remains unexplored.

The NHANES provides a unique opportunity to investigate this scientific question due to its comprehensive assessment of urinary arsenic concentrations, detailed dietary intake data, and nationally representative sampling design (20). Previous analyses of NHANES data have demonstrated associations between urinary arsenic levels and various health outcomes, including cancer prevalence (21). Nonetheless, no study has examined the potential modifying effect of a gut microbiota-oriented dietary index on the arsenic bladder cancer relationship.

This study aims to address this knowledge gap by investigating the potential interaction between urinary arsenic exposure and DIGM on bladder cancer risk across multiple cycles of NHANES. We hypothesized that a higher DIGM score indicating a diet more conducive to a healthy gut microbiome would attenuate the positive association between urinary arsenic concentration and the prevalence of bladder cancer. Understanding this effect modification could inform targeted dietary interventions for populations with elevated arsenic exposure and provide insights into the complex interplay between environmental exposures, diet, gut microbiome, and cancer development.

## 2 Materials and methods

### 2.1 Study population and design

**FIGURE 1:**
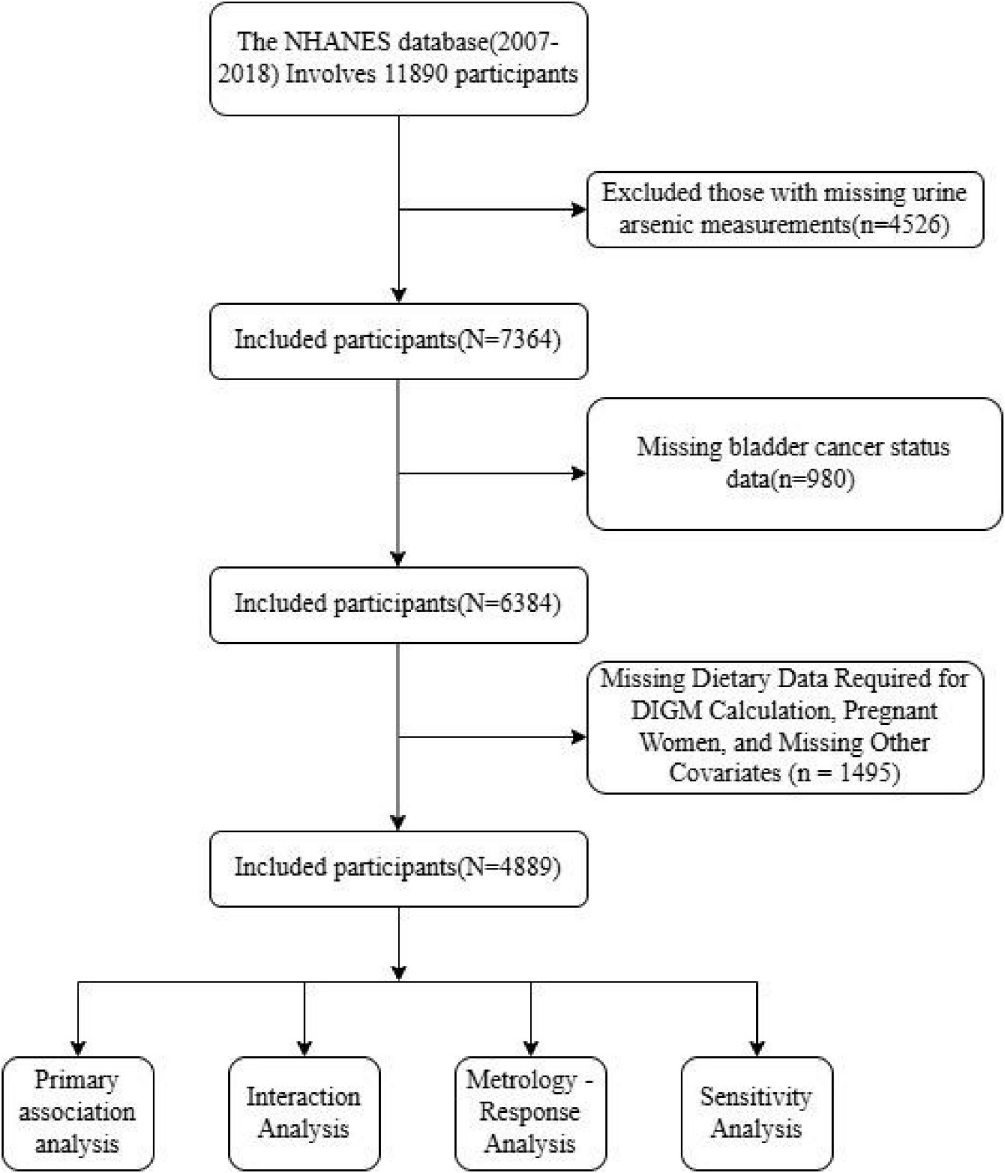
Study flow chart.

We analyzed data from six consecutive 2-year cycles of NHANES (2007–2008 to 2017–2018). NHANES employs a complex, multistage probability sampling design to obtain a nationally representative sample of the non-institutionalized U.S. civilian population. Our initial sample consisted of 11,890 participants. We excluded individuals with missing urinary arsenic measurements (*n* = 4,526), those with missing bladder cancer status (*n* = 980), participants lacking dietary data required for DIGM calculation (*n* = 0), pregnant women (*n* = 0), and individuals with missing data on other covariates (*n* = 1,495). The final analytical sample included 4,889 participants (Figure 1). The NHANES protocol was approved by the Research Ethics Review Board of the National Center for Health Statistics (NCHS), and all participants provided written informed consent.

### 2.2 Assessment of bladder cancer

Bladder cancer status was ascertained using the Medical Conditions Questionnaire in NHANES, which was administered via a computer-assisted personal interview system. In accordance with the study protocol, two key questions were used to identify bladder cancer cases. First, all participants were asked: “Has a doctor or other health professional ever told you that you had cancer or a malignancy of any kind?” Those who responded “yes” were then asked: “What kind of cancer was it?” Participants could report up to three different types of cancer. Those who indicated “bladder cancer” were defined as bladder cancer cases. Participants reporting multiple cancers were classified as bladder cancer-positive if bladder cancer was among the reported malignancies. Individuals who reported a history of cancer but not bladder cancer were categorized as having another cancer type and were excluded in certain sensitivity analyses. It is important to note that NHANES does not collect detailed information on cancer diagnosis timing, stage, or treatment, which constitutes a limitation of this study (22).

### 2.3 Measurement of urinary arsenic

Urine samples were collected at NHANES mobile examination centers using standardized protocols and stored at –20°C until analysis. Measurements of total urinary arsenic and its metabolites were performed by the NHANES contract laborator using high-performance liquid chromatography coupled with inductively coupled plasma dynamic reaction cell mass spectrometry. The variable URXUAS was used as the primary exposure metric. For concentrations below the limit of detection, we used the substitution value provided by NHANES (LOD/√2). To account for urine dilution, urinary creatinine was included as a covariate in statistical models. For analysis, urinary arsenic concentrations were log-transformed to approximate a normal distribution and used as a continuous variable. In some analyses, arsenic levels were also categorized into quartiles to evaluate potential non-linear relationships.

### 2.4 Construction of diet index for intestinal microflora

The DIGM was constructed based on the scoring system established by Kase et al., which incorporates 14 food items and nutrients (23). Beneficial components included avocados, broccoli, chickpeas, coffee, cranberries, fermented dairy products, dietary fiber, soy, and whole grains. Green tea was excluded due to the lack of specific tea type classification in NHANES. Detrimental components consisted of red meat, processed meat, refined grains, and a high-fat diet, defined as ≥ 40% of total energy intake from fat. DIGM scores were derived from dietary recall data in NHANES 2007–2020. For beneficial foods, one point was assigned if intake was equal to or above the sex-specific median; otherwise, zero points were given. For detrimental dietary components, a score of zero was assigned if consumption reached or exceeded the sex-specific median (or 40% for high-fat diet), and one point was given otherwise. The total DIGM score was calculated by summing all component scores, yielding a theoretical range of 0–13. This sex-specific classification approach was adopted for several reasons. First, it aligns with the methodology established in the original DIGM validation study in the NHANES population (24). Second, substantial evidence demonstrates significant sex-based differences in gut microbiota composition and function (25, 26), which may influence how dietary factors reflected in DIGM scores impact health outcomes. Third, well-documented sex differences in dietary patterns and nutritional requirements (27) suggest that sex-specific thresholds may more accurately classify relative dietary quality affecting gut microbiota. This approach is consistent with established methodological practices in nutritional epidemiology when analyzing dietary indices in relation to health outcomes.

For analysis, the total DIGM score was categorized into sex-specific tertiles (T1: low; T2: medium; T3: high) to evaluate its potential effect modification on the association between urinary arsenic and bladder cancer. We also explored the effects of DIGM as a continuous variable.

### 2.5 Covariates

A range of covariates were considered in this study, encompassing demographic characteristics, socioeconomic status, personal behaviors, and health conditions. These included age, sex, race/ethnicity, educational attainment, marital status, and household income. Individual metrics such as BMI, smoking status, and alcohol consumption were also incorporated. Smoking status was categorized into three groups: never smokers (fewer than 100 cigarettes in lifetime), former smokers (more than 100 cigarettes in lifetime but not currently smoking at all), and current smokers (more than 100 cigarettes in lifetime and currently smoking some days or every day). Alcohol consumption was classified based on intake levels: never drinkers (fewer than 12 drinks in lifetime), former drinkers (consumed 12 or more drinks in 1 year in the past but not in the last year, or never drank in the last year but had 12 or more drinks in lifetime), heavy drinkers (women consuming ≥ 3 drinks per day or men ≥ 4 drinks per day, or binge drinking on more than 5 occasions per month), moderate drinkers (women consuming ≥ 2 drinks per day or men ≥ 3 drinks per day, or binge drinking on at least 2 occasions per month), and light drinkers (those not meeting any of the above criteria). Health conditions included hypertension, defined as systolic blood pressure ≥ 130 mm Hg, diastolic blood pressure ≥ 80 mm Hg, or current use of antihypertensive medication; and diabetes, defined as self-reported physician diagnosis or use of glucose-lowering medication. Additional laboratory measures were also included as control variables in the models.

### 2.6 Statistical analysis

All analyses accounted for the complex survey design of NHANES incorporating examination sample weights, strata, and primary sampling units in accordance with NCHS guidelines to produce nationally representative estimates. Weighted multivariable logistic regression models were used to evaluate the association between urinary arsenic concentration and bladder cancer, as well as the potential modifying effect of the DIGM. The following analytical steps were performed: Primary Association Analysis: We first examined the overall association between urinary arsenic and bladder cancer. Urinary arsenic was included as a continuous variable (log-transformed), with results expressed as the effect estimate. It was also categorized into quartiles to assess potential non-linear relationships. Covariate selection was based on a causal framework consideration of directed acyclic graphs (DAGs) designed to control for confounding while avoiding overadjustment. We divided covariates into three categories: (1) common cause variables (e.g., age, sex) that may affect both exposure and outcome and must be adjusted to reduce confounding; (2) exposure-related variables (e.g., BMI) that may affect arsenic metabolism but are not directly associated with outcome; and (3) potential mediating variables (e.g., diabetes and cardiovascular disease) that may be on the causal path of exposure to outcome. We used a hierarchical adjustment approach to develop the following model: Model 1: unadjusted covariates. Model 2: Age, Sex, and Race adjusted. Model 3: Adjusted for Age, Sex, Race, BMI, Hypertension, Diabetes, CVD, Creatinine, UA, BUN, and HbA1c. This approach allowed us to assess the association of arsenic exposure on bladder cancer while taking into account the overadjustment bias that could result.

To assess effect modification, multiplicative interaction terms (urinary arsenic*DIGM tertile) were introduced into the fully adjusted model (Model 3). The statistical significance of interactions was evaluated using Wald tests. If a significant interaction was detected, stratified analyses were conducted by DIGM tertile to estimate the association between urinary arsenic and bladder cancer within each stratum. Dose-Response Analysis: Restricted cubic splines (RCS) with three knots (at the 10th, 50th, and 90th percentiles) were used to explore potential non-linear dose-response relationships between urinary arsenic and bladder cancer. Additive Interaction Assessment: Measures of additive interaction including the relative excess risk due to interaction (RERI), the attributable proportion (AP), and the synergy index (S) were calculated to evaluate interaction on a public health scale.

To test the robustness of the findings, the following sensitivity analyses were conducted:(1) Excluding participants with a history of other cancers;(2) Replacing covariate-adjusted urinary creatinine with creatinine-standardized urinary arsenic values;(3) Using the full survey weight instead of the urine subsample weight to evaluate potential selection bias. All statistical analyses were performed using R software (version 4.3.1) with the survey and rms packages. Two-sided *p*-values < 0.05 were considered statistically significant.

## 3 Results

### 3.1 Participant characteristics

A total of 4,889 participants were included in this study, among whom 585 (11.96%) had bladder cancer. Participants in the bladder cancer group were significantly older than those in the non-cancer group (mean age: 52.96 vs. 46.73 years, *p* < 0.0001) and had a higher body mass index (30.10 vs. 28.83, *p* < 0.001). The bladder cancer group also had a significantly higher proportion of males (58.14 vs. 49.30%), non-Hispanic White individuals (78.80 vs. 71.22%), and married participants (62.77 vs. 53.20%). Metabolic profiling revealed significantly elevated levels of HbA1c, creatinine, uric acid, and blood urea nitrogen in the bladder cancer group, along with lower estimated glomerular filtration rate (eGFR) (all *p* < 0.001). Additionally, the prevalence of hypertension (50.19 vs. 36.73%) and diabetes (24.94 vs. 12.87%) was significantly higher in the bladder cancer group (all *p* < 0.001) (Table 1).

**TABLE 1:** Comparison of general characteristics between bladder cancer and non-bladder cancer patients.

Variables	Total (*n* = 4889)	Non-bladder cancer (*n* = 4,304)	Bladder cancer (*n* = 585)	*P*-value
Age, mean (SE)	47.47(0.50)	46.73(0.51)	52.96(0.77)	< 0.001
PIR, mean (SE)	2.92(0.05)	2.92(0.05)	2.94(0.12)	0.860
BMI, mean (SE)	28.98(0.14)	28.83(0.15)	30.10(0.30)	<0.001
DIGM, mean (SE)	4.68(0.04)	4.69(0.04)	4.62(0.11)	0.580
HbA1c, mean (SE)	5.66(0.02)	5.64(0.02)	5.84(0.05)	<0.001
Creatinine, mean (SE)	77.97(0.49)	77.25(0.50)	83.29(1.45)	<0.001
UA, mean (SE)	322.63(2.10)	319.47(2.24)	346.17(3.68)	<0.001
BUN, mean (SE)	4.75(0.04)	4.68(0.05)	5.28(0.13)	<0.001
eGFR, mean (SE)	94.34(0.56)	95.34(0.55)	86.87(1.18)	<0.001
Urine arsenic, mean (SE)	7.83(0.31)	6.81(0.16)	15.40(1.66)	<0.001
Sex, %(SE)				0.010
Female	49.66(0.02)	50.70(1.07)	41.86(2.76)	
Male	50.34(0.02)	49.30(1.07)	58.14(2.76)	
Race, %(SE)				<0.001
Mexican American	7.59(0.01)	8.02(0.95)	4.38(0.98)	
Non-Hispanic black	9.64(0.01)	10.24(0.97)	5.13(1.04)	
Non-Hispanic white	72.12(0.04)	71.22(1.77)	78.80(2.52)	
Other	10.65(0.01)	10.51(0.77)	11.69(1.76)	
Marital, %(SE)				< 0.001
Divorced	11.60(0.01)	11.47(0.69)	12.54(1.75)	
Married	54.34(0.02)	53.20(1.38)	62.77(2.41)	
Never married	17.33(0.01)	18.35(1.20)	9.72(1.41)	
Other	16.74(0.01)	16.98(0.76)	14.97(1.68)	
Education, %(SE)				0.918
High school or equivalent	26.39(0.01)	26.40(1.16)	26.37(2.60)	
Less than high school	16.17(0.01)	16.17(0.94)	16.15(1.78)	
Some college or above	57.44(0.02)	57.43(1.49)	57.47(3.32)	
Smoke, %(SE)				0.080
Former	23.76(0.01)	23.11(1.15)	28.66(2.38)	
Never	49.65(0.02)	49.94(1.25)	47.53(3.13)	
Now	26.58(0.01)	26.95(1.11)	23.81(2.53)	
Alcohol, %(SE)				<0.001
Former	16.69(0.01)	16.10(0.79)	21.05(2.91)	
Heavy	23.25(0.01)	24.17(1.03)	16.39(2.03)	
Mild	34.49(0.02)	33.61(1.22)	41.05(2.99)	
Moderate	17.33(0.01)	17.68(0.90)	14.67(1.81)	
Never	8.24(0.01)	8.43(0.68)	6.84(0.99)	
Hypertension,%(SE)				<0.001
No	61.67(0.02)	63.27(1.16)	49.81(3.01)	
Yes	38.33(0.02)	36.73(1.16)	50.19(3.01)	
Diabetes,%(SE)				<0.001
Borderline	8.15(0.01)	8.40(0.57)	6.27(1.38)	
No	77.55(0.03)	78.73(0.86)	68.79(2.83)	
Yes	14.30(0.01)	12.87(0.70)	24.94(2.41)	
CVD, %(SE)				<0.001
No	91.20(0.03)	91.91(0.64)	85.93(1.63)	
Yes	8.80(0.01)	8.09(0.64)	14.07(1.63)	

DIGM, Dietary Index for Gut Microbiota; BMI, Body mass index; PIR, Poverty income ratio; HbA1c, Glycosylated hemoglobin; UA, Uric acid; BUN, Blood urea nitrogen; eGFR, Estimated glomerular filtration rate; CVD, Cardiovascular disorders.

### 3.2 Association of urinary arsenic with bladder cancer

Table 2 presents the results of the multivariable logistic regression analysis examining the association between urinary arsenic concentration and bladder cancer. In the fully adjusted model (Model 3), each unit increase in urinary arsenic was significantly associated with an elevated risk of bladder cancer (OR = 1.07, 95% CI: 1.06–1.09, *p* < 0.001). When urinary arsenic was categorized into quartiles, a dose-response relationship was observed. Compared with the lowest quartile (Q1), higher quartiles of urinary arsenic were associated with progressively increased risks of bladder cancer. Specifically, the highest quartile (Q4) showed a significantly elevated risk (OR = 2.22, 95% CI: 1.53–3.22, *p* < 0.001), while Q2 and Q3 exhibited smaller and marginally significant effects.

**TABLE 2:** Multivariate logistic regression analysis of the association between urinary arsenic concentration and bladder cancer risk.

Variables	Model 1		Model 2		Model 3	
	OR (95%CI)	*P*	OR (95%CI)	*P*	OR (95%CI)	*P*
Urine arsenic (μg/g Cr)	1.08(1.06,1.09)	< 0.0001	1.07(1.06,1.09)	< 0.0001	1.07(1.06,1.09)	< 0.0001
**Urine arsenic (μg/g Cr)Q**						
Q1	Ref	Ref	Ref	Ref	Ref	Ref
Q2	1.23(0.89,1.72)	0.21	1.28(0.91,1.81)	0.15	1.28(0.92,1.78)	0.14
Q3	1.15(0.84,1.57)	0.39	1.20(0.86,1.66)	0.27	1.21(0.87,1.68)	0.25
Q4	2.25(1.58,3.19)	< 0.0001	2.27(1.58,3.24)	< 0.0001	2.22(1.53,3.22)	< 0.0001
P for trend	<0.0001		<0.0001		< 0.001	

OR, Odds ratio; CI, confidence interval; Ref, reference. Model 1: No adjustments made; Model 2: Adjusted for Age, Sex, Race; Model 3: Adjusted for Age, Sex, Race, BMI, Hypertension, Diabetes, CVD, UA, BUN, HbA1c and Creatinine.

**FIGURE 2:**
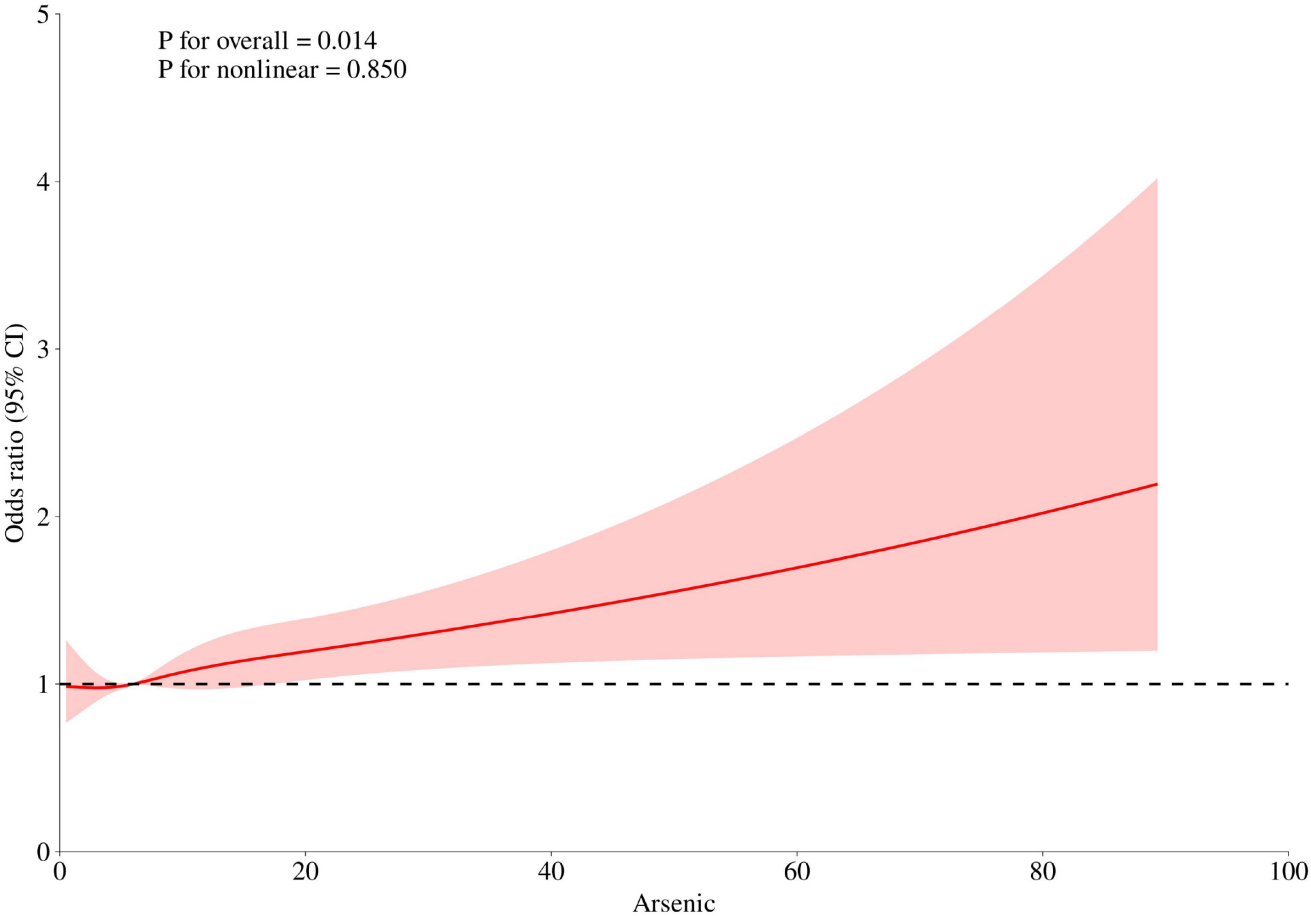
Restricted cubic spline analysis. Adjusted for Age, Sex, Race, BMI, Hypertension, Diabetes, CVD, UA, BUN, HbA1c, and Creatinine.

The restricted cubic spline analysis further confirmed a positive dose-response relationship between urinary arsenic concentration and bladder cancer risk, as illustrated in Figure 2.

### 3.3 Modifying effect of DIGM on the association between urinary arsenic and bladder cancer

A significant interaction between urinary arsenic and DIGM tertiles (p for interaction = 0.022) was detected in the fully adjusted model, prompting stratified analysis as presented in Table 3. As shown in Table 3, the association between urinary arsenic and bladder cancer risk was evaluated across DIGM tertiles. In the lowest DIGM tertile (T1, representing the least gut-friendly dietary pattern), each unit increase in urinary arsenic was significantly associated with higher bladder cancer risk (OR = 1.95, 95% CI: 1.46–2.61, *p* < 0.001). This association attenuated in the middle tertile (T2, OR = 1.42, 95% CI: 1.03–1.96, *p* = 0.034), and became non-significant in the highest tertile (T3, representing the most gut-friendly dietary pattern, OR = 1.13, 95% CI: 0.89–1.44, *p* = 0.301).

**TABLE 3:** Stratified analysis of the association between urinary arsenic and bladder cancer risk: grouped by tertile of the DIGM.

Urine arsenic exposure (μg/g Cr)	DIGM T1-Low OR (95% CI)	*P*	DIGM T2-Med OR (95% CI)	*P*	DIGM T3-High OR (95% CI)	*P*
Continuous	1.95 (1.46–2.61)	< 0.001	1.42 (1.03–1.96)	0.034	1.13 (0.89–1.44)	0.301
**Category (quartile)**						
Q1	Ref	Ref	Ref	Ref	Ref	Ref
Q2	1.58 (0.82–3.05)	0.237	1.32 (0.72–2.43)	0.356	1.09 (0.61–1.94)	0.208
Q3	2.16 (1.17–4.01)	0.038	1.47 (0.82–2.64)	0.421	1.15 (0.66–2.02)	0.306
Q4	2.97 (1.63–5.42)	< 0.001	1.86 (1.04–3.30)	0.041	1.25 (0.72–2.18)	0.427
P for trend	< 0.001		0.028		0.418	

Interaction between urinary arsenic and DIGM tertiles was significant (p for interaction = 0.022) in the fully adjusted model. Adjusted for Age, Sex, Race, BMI, Hypertension, Diabetes, CVD, UA, BUN, HbA1c and Creatinine. CI, Confidence Interval; OR, Odds Ratio; Ref, Reference; DIGM, Dietary Index for Gut Microbiota.

This modifying effect was more pronounced when urinary arsenic was analyzed by quartiles. In the T1 DIGM group, participants in the highest quartile (Q4) of urinary arsenic had nearly three times the risk of bladder cancer compared to those in the lowest quartile (Q1) (OR = 2.97, 95% CI: 1.63–5.42, *p* < 0.001). By contrast, in the T3 DIGM group, the association between the highest quartile of urinary arsenic and bladder cancer risk was no longer significant (OR = 1.25, 95% CI: 0.72–2.18, *p* = 0.427).

**FIGURE 3:**
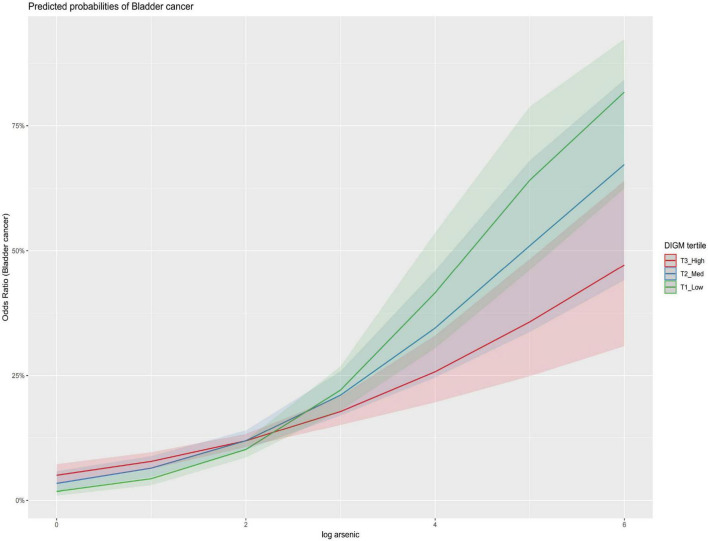
Forest plot for association between urinary arsenic and bladder cancer risk in different DIGM tertiles. Adjusted for Age, Sex, Race, BMI, Hypertension, Diabetes, CVD, UA, BUN, HbA1c, and Creatinine.

**FIGURE 4:**
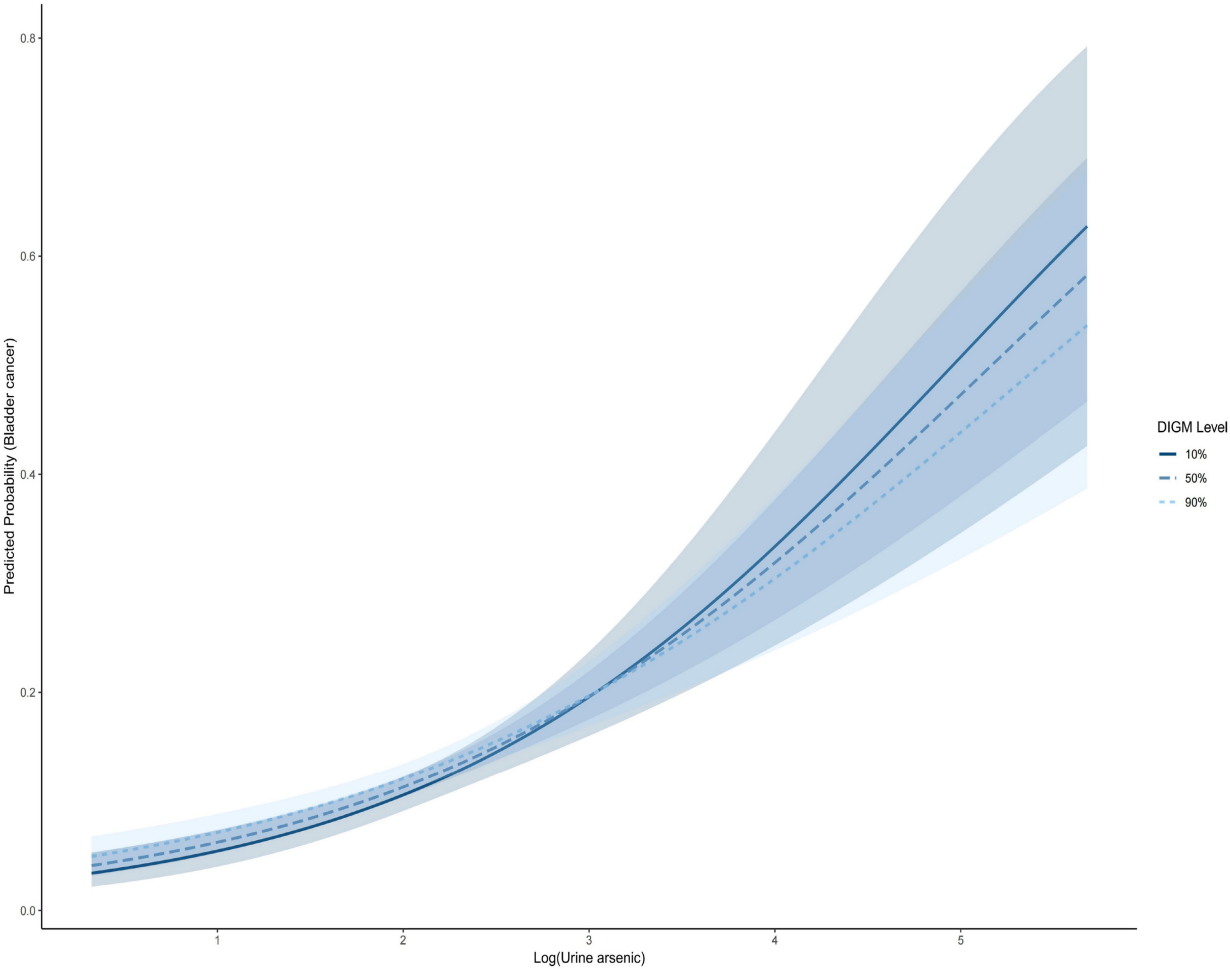
Restricted cubic splines demonstrated dose-response relationships between urinary arsenic and bladder cancer at different DIGM levels. Adjusted for Age, Sex, Race, BMI, Hypertension, Diabetes, CVD, UA, BUN, HbA1c, and Creatinine.

Figure 3 presents a forest plot illustrating the association between urinary arsenic and bladder cancer risk across DIGM tertiles. A clear pattern was observed: as the DIGM score increased from the lowest to the highest tertile, the effect estimate of urinary arsenic gradually decreased, suggesting that a gut-microbiota-friendly diet may exert a protective effect against arsenic-related bladder cancer risk. Figure 4 displays the dose-response relationships between urinary arsenic and bladder cancer risk stratified by DIGM levels, using restricted cubic splines. In the low DIGM group, a distinct positive non-linear association was observed, with risk accelerating at higher urinary arsenic concentrations. In contrast, the dose-response curve in the high DIGM group was nearly flat, indicating that a higher DIGM score may attenuate the impact of arsenic exposure on bladder cancer risk.

### 3.4 Assessment of additive interactions

Analysis of additive interaction indicated a synergistic effect between urinary arsenic exposure and low DIGM score. The RERI was 0.47 (95% CI: 0.08–0.86), indicating excess risk attributable to their interaction. The AP was 0.31 (95% CI: 0.06–0.56), suggesting that approximately 31% of the excess risk associated with joint exposure was due to interaction. The synergy index was 1.63 (95% CI: 1.09–2.42), further supporting a synergistic relationship between the two factors (Table 4).

**TABLE 4:** Assessment of additive interaction between urinary arsenic exposure and low DIGM score on bladder cancer risk.

Interaction measures	OR (95% CI)	*P*-value
RERI	0.47 (0.08, 0.86)	0.018
AP	0.31 (0.06, 0.56)	0.015
S	1.63 (1.09, 2.42)	0.017

CI, Confidence Interval; OR, Odds Ratio; RERI, Relative excess risk; AP, Attributable ratio; S, Synergy index. Adjusted for Age, Sex, Race, BMI, Hypertension, Diabetes, CVD, UA, BUN, HbA1c and Creatinine.

### 3.5 Sensitivity analysis

Our primary findings remained robust across various sensitivity analyses. After excluding participants with a history of other cancers, the results were marginally strengthened. Analyses using creatinine-standardized urinary arsenic concentrations yielded results consistent with the main findings. The use of the full survey weight, rather than the urine subsample weight, produced estimates similar to those in the primary analysis, further enhancing the credibility of our results (Table 5).

**TABLE 5:** Sensitivity analysis of the association between urinary arsenic and bladder cancer risk.

Sensitivity analysis	Main model OR (95% CI)	*P*-value	P for interaction (Urine arsenic*DIGM)
Primary analysis	1.37 (1.12–1.68)	0.002	0.022
Exclude other cancer patients	1.41 (1.15–1.73)	0.001	0.018
Urine arsenic creatinine corrected (μg/g Cr)	1.35 (1.10–1.66)	0.004	0.025
Use full survey weights	1.34 (1.09–1.65)	0.006	0.028

CI, Confidence Interval; OR, Odds Ratio. Adjusted for Age, Sex, Race, BMI, Hypertension, Diabetes, CVD, UA, BUN, HbA1c and Creatinine.

## 4 Discussion

This study is the first to reveal a significant modifying effect of the DIGM on the association between urinary arsenic concentration and bladder cancer risk, providing important evidence at the intersection of environmental toxicology and nutritional epidemiology. We observed a clear dose-response relationship between arsenic exposure and bladder cancer risk, which was strongly influenced by dietary patterns. This suggests that dietary interventions may represent a potential strategy for mitigating arsenic-related health risks.

Our demographic findings align with established bladder cancer epidemiology. The higher prevalence among older participants, males, and non-Hispanic White individuals reflects well-documented patterns in bladder cancer incidence (28). Age-related risk likely stems from cumulative carcinogen exposure and diminished DNA repair capacity (28), while the male predominance (approximately 3:1 in our cohort) may result from both differential exposures and hormonal factors, with androgen receptor signaling potentially promoting bladder cancer development (29). The elevated risk observed in participants with higher BMI is consistent with emerging evidence linking obesity to bladder cancer through inflammatory pathways and altered metabolism of environmental toxicants (30).

The demographic variations observed in our study may interact with arsenic exposure effects. For instance, the higher prevalence among non-Hispanic White participants mirrors documented racial disparities in bladder cancer incidence, though mortality patterns often differ across racial groups (31). Similarly, the unexpected finding of higher prevalence among married individuals may reflect detection bias through spousal encouragement for medical care rather than true risk elevation (32). These patterns suggest that demographic factors may modify the relationship between arsenic metabolism efficiency and bladder cancer susceptibility, highlighting the importance of considering individual characteristics when assessing environmental exposure risks.

The positive association between urinary arsenic and bladder cancer risk (OR = 2.22, 95% CI: 1.53–3.22 for the highest vs. lowest quartile) is consistent with previous studies. A case-control study by Ferreccio et al. in a high-arsenic region of Chile showed that arsenic-related bladder cancer risk remained significantly elevated even decades after exposure cessation (33). A multicenter study by Baris et al. also confirmed that chronic low-level arsenic exposure is associated with increased bladder cancer incidence (34). Notably, the strength of the association observed in our U.S. representative population was comparable to effect sizes reported by Martinez et al. in high-exposure regions (35), indicating that arsenic exerts carcinogenic effects on the bladder epithelium even at relatively low exposure levels. The restricted cubic spline analysis further supported the non-linear nature of this relationship, consistent with the threshold-effect hypothesis proposed by Cohen et al. regarding arsenic carcinogenesis (36).

The most innovative finding of this study is the significant modifying effect of DIGM on the arsenic bladder cancer association. The association was strongest among individuals with low DIGM scores (OR = 1.95, 95% CI: 1.46–2.61), while it was attenuated to non-significance in those with high DIGM scores (OR = 1.13, 95% CI: 0.89–1.44). This finding advances beyond traditional environmental epidemiology models focused on single exposures by highlighting the crucial regulatory role of host factors particularly diet and the microbiome in environmental carcinogenesis. Although a prospective study by Argos et al. in Bangladesh found that dietary folate intake mitigated arsenic-related skin lesion risk (37), our study is the first to examine the moderating effect of diet on arsenic toxicity from the perspective of overall dietary patterns and gut microbial ecology.

Notably, our analysis revealed that the association between urinary arsenic and bladder cancer remained remarkably stable across progressively adjusted models. The odds ratios changed minimally from the unadjusted Model 1 to the fully adjusted Model 3, which included numerous potential confounders such as age, sex, race, BMI, hypertension, diabetes, cardiovascular disease, and multiple biomarkers. This stability of effect estimates warrants further discussion.

The persistence of the association despite extensive covariate adjustment suggests several important implications. First, it indicates that urinary arsenic may have a strong and independent relationship with bladder cancer risk, consistent with its classification as a Group 1 carcinogen by the International Agency for Research on Cancer (38). The minimal attenuation of effect estimates after adjustment reinforces the biological plausibility of arsenic’s direct carcinogenic effects on bladder tissue, which operates through mechanisms including oxidative stress, genotoxicity, and interference with DNA repair pathways (39, 40). Second, the stability of effect estimates suggests that the relationship between arsenic exposure and bladder cancer may not be substantially confounded by the measured covariates in our study. Similar phenomena have been observed in other studies examining established carcinogen-cancer relationships (41, 42). For instance, García-Esquinas et al. (43) reported comparable stability in effect estimates when examining arsenic exposure and cancer outcomes in another large population study, suggesting that when strong carcinogenic effects are present, they often persist despite extensive statistical adjustment. Third, while our models accounted for numerous important covariates, the stability of estimates might also reflect the presence of residual confounding by unmeasured variables that correlate with both arsenic exposure and bladder cancer risk. However, the consistency of our findings with established biological mechanisms and previous epidemiological studies supports the validity of the observed association. Finally, from a methodological perspective, the stability of effect estimates across models enhances our confidence in the robustness of the association. As demonstrated by Greenland and colleagues (44), when effect estimates remain stable despite increasing levels of adjustment, it often indicates that the primary exposure-outcome relationship is less vulnerable to bias from measured confounders, particularly when the direction and magnitude remain consistent.

The gut microbiota may influence arsenic metabolism and toxicity through several mechanisms. First, specific bacteria can directly participate in the biotransformation of arsenic. Studies have shown that bacterial arsenic resistance genes can catalyze methylation, reduction, and sulfidation of arsenic, altering its bioavailability and toxicity (45). Rubin et al. demonstrated that bacteria such as Clostridium and Bacteroides can convert inorganic arsenic into less toxic thioarsenates (46). Second, microbial metabolites may modulate host arsenic metabolic capacity. Kumana et al. reported that microbial-derived sulfides provide sulfhydryl groups that participate in arsenic methylation, enhancing detoxification (47). Third, a healthy gut microbiome helps maintain intestinal barrier integrity, potentially reducing arsenic absorption. Laue et al. found that probiotic supplementation significantly alleviated arsenic-induced increases in gut permeability and inflammatory responses (48).

A high-DIGM diet is typically rich in dietary fiber, polyphenols, and prebiotics, which promote beneficial microbial growth and enhance gut health. Research indicates that diets high in resistant starch significantly increase the abundance of Bifidobacterium and Ruminococcus, while reducing inflammatory markers (49). Serrano et al. found that high-fiber diets promote short-chain fatty acid production, improve gut barrier function, and reduce endotoxin leakage (50). These mechanisms may collectively explain the protective effect of high DIGM scores against the arsenic bladder cancer relationship.

Our additive interaction analysis further supports a biologically meaningful synergistic effect between arsenic exposure and DIGM. A RERI of 0.47 (95% CI: 0.08–0.86) and an attributable proportion of 0.31 (95% CI: 0.06–0.56) indicate that approximately 31% of the excess risk from joint exposure is attributable to their interaction. This pattern aligns with the “multiple-hit” carcinogenesis model proposed by Knudsen et al., wherein environmental toxicants and host susceptibility factors interact to promote tumor development (51). Similarly, Neamat-Allah et al. reported significant interactions between dietary patterns and environmental pollutant exposure in the development of chronic diseases (52).

The modifying effect of dietary patterns on arsenic-induced bladder cancer risk observed in this study may be closely related to trace element homeostasis and oxidative stress regulation. Recent research has increasingly focused on the central role of trace element balance in regulating epithelial cell oxidative stress and metabolic responses, which directly affects susceptibility to various environment-related cancers including bladder cancer (53). Notably, certain micronutrients (such as selenium) may influence arsenic metabolism and toxicity through interactions with arsenic or by regulating antioxidant enzyme activities. Concurrently, rapid developments in the field of biomarkers, especially innovations in liquid biopsy technology, provide new directions for future in-depth research. Novel biomarkers such as circulating tumor DNA and exosomes enable non-invasive, dynamic monitoring of early carcinogenic processes and molecular characteristics induced by environmental exposures (54). Combining these advanced biological monitoring tools with dietary exposure assessment will help more precisely reveal the interaction mechanisms among gut microbiota, diet, and arsenic exposure, promoting the development of individualized risk assessment and precision prevention strategies.

From a public health perspective, our findings carry important implications. According to the World Health Organization, over 200 million people worldwide are exposed to arsenic-contaminated drinking water, and complete elimination of this exposure remains technically and economically challenging. Our study suggests that improving dietary structure to promote gut microbial health may represent a cost-effective intervention strategy to reduce the burden of arsenic-related diseases. A dietary intervention study by Yu et al. showed that a diet rich in probiotics and prebiotics significantly reduced blood arsenic levels and improved oxidative stress markers (55). Oremland et al. also highlighted the potential of microbial remediation strategies to mitigate the effects of environmental arsenic contamination (56).

The robustness of our primary findings was confirmed through sensitivity analyses. Analyses using creatinine-standardized urinary arsenic concentrations yielded results consistent with the main outcomes, which aligns with findings by Nermell et al. that arsenic measurement adjustment methods do not substantially alter its association with health endpoints (57). The slight strengthening of results after excluding participants with a history of other cancers may reflect the influence of cancer treatments on arsenic metabolism and microbiome composition, as reported by Byun et al. (58). The consistency of results across different weighting approaches further enhances the credibility of our findings.

Despite several strengths including a large sample size, national representativeness, standardized exposure assessment, and comprehensive control for confounders this study has some limitations. Firstly, the cross-sectional design of our study significantly limits causal and temporal inference. Without longitudinal data, we cannot determine whether the observed association precedes disease development. Of particular note, patients may change their eating habits and health behaviors after cancer diagnosis, which may influence DIGM scores and arsenic metabolism profiles and thus confound our observations. For example, patients may have adopted a healthier diet after diagnosis, changing the key variables we measured. Future prospective cohort studies that track dietary patterns and arsenic metabolism changes before and after diagnosis are needed to verify the directionality of these relationships. Secondly, although urinary arsenic is a well-established biomarker, a single measurement mainly reflects short-term exposure (arsenic has a half-life of about 4 days in the human body) and cannot accurately represent long-term cumulative doses, which are more relevant to the carcinogenic process. This limitation is particularly important given that the latency period from environmental carcinogen exposure to bladder cancer development typically spans decades. In addition, urinary arsenic concentrations can be influenced by daily water intake, dietary patterns, and individual metabolic differences. Future studies should consider surrogate biomarkers such as long-term repeated measurements or adoption of arsenic content in fingernails, hair that may better reflect chronic exposure and thus more accurately assess the relationship between long-term arsenic burden and cancer risk. Thirdly, the DIGM, as a proxy for gut microbial health, has inherent limitations. Although we adjusted for a wide range of potential confounders, unmeasured confounding may remain. Additionally, reliance on self-reported bladder cancer diagnosis may introduce misclassification. Future studies should investigate the modifying effects of specific dietary components within the DIGM to develop more targeted intervention strategies. Further research is also needed to elucidate the molecular mechanisms underlying the triad of arsenic microbiome host interactions. Moreover, given variations in arsenic exposure sources and metabolic patterns across populations, it is important to examine the consistency of the DIGM modification effect in diverse racial and regional groups.

## 5 Conclusion

In conclusion, this study is the first to identify that the DIGM may modify the association between urinary arsenic exposure and bladder cancer risk. This observation suggests that dietary patterns might modulate arsenic metabolism and toxicity through influences on gut microbial ecology, although our study did not directly measure the microbiome. Based on previous literature, we hypothesize that diets supporting healthy gut microbiota may impact arsenic biotransformation, absorption, and excretion through various pathways, but these mechanisms require validation through direct microbiome analysis in future research. This preliminary finding provides potential dietary intervention directions for arsenic-exposed populations, with promise for development into a practical and economical supplementary approach to mitigate health impacts of environmental toxicants, potentially holding public health significance especially in high arsenic exposure areas. Subsequent studies should combine dietary assessment with microbiome measurements to confirm these hypothesized mechanisms and identify optimal intervention strategies.

## Data Availability

The original contributions presented in the study are included in the article/supplementary material, further inquiries can be directed to the corresponding author.
